# Association of Evaluated Glomerular Filtration Rate and Incident Diabetes Mellitus: A Secondary Retrospective Analysis Based on a Chinese Cohort Study

**DOI:** 10.3389/fmed.2021.724582

**Published:** 2022-01-31

**Authors:** Zihe Mo, Haofei Hu, Xiaoqing Du, Qingli Huang, Ping Chen, Linjing Lai, Zhiqun Yu

**Affiliations:** ^1^Department of Physical Examination, DongGuan Tungwah Hospital, Dongguan, China; ^2^Department of Nephrology, The First Affiliated Hospital of Shenzhen University, Shenzhen, China; ^3^Department of Nephrology, Shenzhen Second People's Hospital, Shenzhen, China

**Keywords:** estimated glomerular filtration rate (eGFR), incident diabetes, non-linearity, Cox proportional hazards regression, cubic spline smoothing

## Abstract

**Background:**

Previous studies have revealed that chronic kidney disease (CKD) is a significant risk factor for insulin resistance and diabetes. However, few studies are on the association between estimated glomerular filtration rate (eGFR) and incident diabetes, especially in the Chinese population with eGFR>60 mL/min·1.73 m^2^. This study explored the relationship between eGFR and incident diabetes in a large cohort in the Chinese community.

**Methods:**

This study was a retrospective cohort study. A total of 1,99,435 adults from Rich Healthcare Group in China were studied, including all medical records for participants who received a health check from 2010 to 2016. The target-independent and target-dependent variables were eGFR measured at baseline, and incident diabetes mellitus appeared during the follow-up. After testing the proportion hypothesis, Cox proportional hazards regression was used to investigate the association between eGFR and incident diabetes. A Cox proportional hazards regression with cubic spline functions and smooth curve fitting (the cubic spline smoothing) was used to identify non-linear relationships between eGFR and the risk of diabetes. Additionally, we also performed subgroup analysis and a series sensitivity analysis. It was stated that the data had been uploaded to the DATADRYAD website.

**Result:**

After adjusting gender, body mass index (BMI), systolic blood pressure (SBP), diastolic blood pressure (DBP), fasting plasma glucose (FPG), total cholesterol (TC), low-density lipoprotein cholesterol (LDL-C), high-density lipoprotein cholesterol (HDL-C), triglyceride (TG), alanine aminotransferase (ALT), aspartate aminotransferase (AST), smoking and drinking status, and family history of diabetes, the result showed that eGFR was negatively associated with incident diabetes [HR = 0.986, 95% CI (0.984, 0.988)]. A non-linear relationship was detected between eGFR and incident diabetes, with an inflection point of eGFR of 98.034 mL/min·1.73 m^2^. The effect sizes and the confidence intervals (Cis) on the left and right sides of the inflection point were 0.998 (0.993, 1.003) and 0.976 (0.972, 0.980), respectively. Subgroup analysis showed a stronger association in the population with FPG <6.1 mmol/L, BMI <24 kg/m^2^, SBP <140 mmHg, DBP <90 mmHg and family history without diabetes. The same trend was also seen in women and the population who never smoke.

**Conclusion:**

Estimated glomerular filtration rate is independently associated with incident diabetes. The relationship between eGFR and incident diabetes is also non-linear. eGFR is strongly related to incident diabetes when eGFR was above 98.034 mL/min·1.73 m^2^.

## Background

Diabetes mellitus is one of the utmost common chronic diseases worldwide. In recent decades, the prevalence of diabetes among Chinese adults has increased significantly (1). According to a large, nationally representative survey of Chinese adults, the estimated overall prevalence of diabetes rose to 10.9% in 2013 (2). Consequently, it is imperative to examine and intervene in the risk factors of diabetes. Diabetes is a debilitating disease that may cause various complications, reducing the quality of life and causing severe socioeconomic impacts. Therefore, the identification of risk factors is essential to prevent diabetes.

Patients with chronic kidney disease (CKD) and diabetes mellitus (DM) have common risk factors (3, 4), which suggests that CKD may increase the risk of diabetes. Estimated glomerular filtration rate (eGFR) is a more straightforward and more useful surrogate indicator that reflects the flow rate of filtrate through the kidney. It has been widely used clinically to diagnose CKD and assess renal function (5). A cardiovascular health study was conducted in 4,680 American participants without diabetes; the results (6) showed that a decrease in eGFR was associated with an increase in insulin resistance. In their research, the average eGFR was 72.2 ml/min per 1.73 m^2^. However, during the 12-year median follow-up period, participants with reduced eGFR did not have an increased risk of diabetes (6). In another cohort study with 864 American participants, the results suggested that the relationship between glomerular filtration rate and the incidence of diabetes was not linear. Within the upper and lower limits of GFR, the risk of diabetes was increased (7). However, most of these studies did not perform subgroup analysis. The relatively small sample size and the regional population are limited to other people who can be generalized. Moreover, previous studies regarding the relationship between eGFR and incident diabetes were still limited in the Chinese people and the populations with eGFR>60 ml/min per 1.73 m^2^. Therefore, this study investigated whether eGFR is independently associated with the onset of diabetes in a large cohort of 32 locations and 11 cities in China.

## Methods

### Data Source and Participants

Data were obtained from the “DATADRYAD” database (www.Datadryad.org). This website allows users to download raw data for free. In accordance with the Dryad Terms of Service, we quoted the Dryad data package in this research (8). Variables included in the database were as follows: body mass index (BMI), gender, age, systolic blood pressure (SBP), diastolic blood pressure (DBP), total cholesterol (TC), triglyceride (TG), high-density lipoprotein cholesterol (HDL-C), low-density lipoprotein cholesterol (LDL-C), fasting plasma glucose (FPG), serum creatinine (Scr), serum urea nitrogen (BUN), aspartate aminotransferase (AST), alanine aminotransferase (ALT), drinking status, smoking status, family history of diabetes, years of follow-up, and occurrence of diabetes during the follow-up (8). According to the CKD-EPI equation, our research added the evaluated glomerular filtration rate (eGFR), calculated based on age, gender, and Scr (9). This new Asian modified CKD-EPI equation could make a more precise GFR estimation for Chinese patients with CKD in general practice, especially in the higher GFR group. The authors of the original research waived the copyright in their data, allowing other researchers to reuse these data without restrictions. Therefore, we could use these data for a secondary analysis without infringing on the rights of the author. Since the research ethics approval was obtained in the original research, the secondary research was no longer needed (8).

The data came from the database provided by China Rich Healthcare Group. The study included that 685,277 participants who underwent health check centers in 32 locations and 11 cities in China between 2010 and 2016 were aged at least 20 years and visited at least two times (Beijing, Nanjing, Shanghai, Suzhou, Changzhou, Shenzhen, Chengdu, Hefei, Guangzhou, Nantong, and Wuhan). The data we obtained from the database had been preliminarily screened (8), and participants were excluded as follows: (1) no available information about gender, height, weight, and baseline fasting blood glucose (2) participants whose visit period was <2 years, (3) extreme BMI values (<15 or >55 kg/m^2^), (4) participants with uncertain diabetes status at follow-up, and (5) participants diagnosed with diabetes at baseline (8). Finally, the analysis by Ying Chen et al. included 211,833 participants (8). The research's inclusion or exclusion criteria and outcome measures were explained explicitly in the previous study (8). Our research further excluded participants with missing values of baseline eGFR (*n* = 11,175) from the analysis cohort. To reduce interference, we excluded outliers in eGFR, which were not included in the range of the means ± three standard deviations (SD) (*n* = 1,223) (10). The final analysis included 1,99,435 subjects (109,690 men and 89,745 women) in this study.

### Measurement of Variables

The design of the retrospective cohort study was documented in the original research (8). To provide readers with a clear understanding of the research process, we outlined the research steps. Participants were asked to fill out a detailed questionnaire every time they visited the health check center, which included lifestyle factors, demographic characteristics, family history of chronic diseases, and personal medical history (8). The trained staff measured the weight, height, and blood pressure of the subjects. When measuring weight, subjects were asked to wear light clothes and no shoes, with an accuracy of 0.1 kg. The height measurement was accurate to 0.1 cm. BMI was calculated by dividing weight (kg) by the square of height (m). Standard mercury sphygmomanometers measured blood pressure. After fasting for at least 10 h at each visit, a fasting venous blood sample was collected. Scr, AST, ALT, TC, TG, HDL-C, and LDL-C were measured on an autoanalyzer (Beckman 5800) (8). The glucose oxidase method was used to measure plasma glucose level on an automatic analyzer (Beckman 5800) (8). The target independent variable was eGFR obtained at baseline. The dependent variable was the diabetes event obtained during follow-up.

Studies using healthcare data were often subjected to have selection or observation biases. It was plausible that older and more ill participants sought more care. Additionally, in those individuals, they were found to have more complete data and thus have more confidence in the assessment of diabetes status by the end of the study period.

### Incident Diabetes

The diagnosis of diabetes was defined as fasting blood glucose >7.00 mmol/L and/or self-reported diabetes during the follow-up. As for the censored times, those participants who developed diabetes were not censored. Only those who were not observed to develop diabetes were censored at the latest follow-up time during the study period (8).

### Statistical Analysis

We first used multiple multivariate imputations to handle the missing data of covariants (excluded exposure and outcome) (11). We created 5 imputed datasets using a mice software package (based on chained equations). Therefore, we created 5 complete data for analysis. The imputation model included gender, age, BMI, DBP, SBP, TC, TG, HDL, LDL, FPG, Scr, BUN, AST, ALT, family history, and drinking and smoking status. In addition, we used sensitivity analysis to identify whether created complete data had significant differences from preimputation data. All results of our studies were based on the imputed datasets and were combined with Rubin's rules.

Next, the participants were stratified into five groups according to the eGFR levels, 60–89 ml/min per 1.73 m^2^ group and then by quartiles of the remaining distribution for those >90 ml/min per 1.73 m^2^ for quartile grouping.

Continuous variables with normal and skewed distribution were expressed as means with standard deviations or medians with interquartile ranges, and categorical variables were expressed as percentages of a specific group. Furthermore, differences between different eGFR groups were tested using ANOVA for normally distributed variables, Kruskal–Wallis H test for skewed variables, and Pearson's chi-square test for categorical variables (12). Follow-up person-years were computed from the baseline interview to the date of the diabetes event or the date of follow-up interview, whichever came first (13). Incidence rates were expressed in cumulative incidence and person-years incidence (14). Survival estimates and time-to-event variables were computed using the Kaplan–Meier method. A log-rank test was used to compare the Kaplan–Meier probability of diabetes-free survival among eGFR groups (15).

The proportion hypothesis was tested first. Then, after meeting the proportion requirement, the Cox proportional hazards regression model was used to estimate the risk ratios (HRs) and 95% confidence intervals (CIs) of incident diabetes. The results of unadjusted, minimally adjusted analyses and fully adjusted analyses are simultaneously shown based on the STROBE statement (16). We defined confounders as variables that changed the hazard ratio estimate for a contraceptive method by 10% or more when included in the model (17).

Moreover, a Cox proportional hazards regression with cubic spline functions and smooth curve fitting (the cubic spline smoothing) were used to address the non-linear association between eGFR and incident diabetes since eGFR was a continuous variable (18). If a non-linearity was detected, we calculated the inflection point using a recursive algorithm. Then, a two-piecewise linear slope was performed to calculate the threshold effect of the eGFR on incident diabetes in terms of the smoothing plot (19). The log-likelihood ratio test was employed to determine the most suitable model for describing the association between eGFR and diabetes risk. Moreover, the Cox proportional hazards models were applied to explore the robustness of the results in various subgroups (gender, age, FPG, BMI, DBP, SBP, smoking and drinking status, and family history). The continuous variable was first converted into a categorical variable based on clinical cut point or tertile. Each stratification adjusted for all the factors (BMI, gender, DBP, SBP, TC, FPG, TG, HDL, LDL, AST, ALT, drinking and smoking status, and family history) except the stratification factor itself. Tests for interaction were performed with the likelihood ratio test of models with and without interaction terms (20, 21).

A series sensitivity analysis was conducted to ensure the robustness of data analysis (22). eGFR was converted into a categorical variable, and the *p*-value was calculated for the trend. The test's purpose was to verify the results of treating eGFR as a continuous variable and determine the possibility of non-linearity. In other sensitivity analyses, we excluded smoking and drinking status from the multivariate model. Because the percentage of missing data on smoking and drinking status was about 70%, this was very high and might not be suitable as covariates adjusted in the model. We also excluded participants with fasting blood glucose >6.1 mmol/L because these participants were more likely to develop diabetes.

All analyses were performed using R (http://www.R-project.org) and EmpowerStats software (www.empowerstats.com, X&Y solutions, Inc., Boston, MA, USA). *p* < 0.05 (two-sided) were considered statistically significant.

## Results

In total, 199,435 participants (45.0% women) were included in the analysis. The mean age was 42.6 ± 12.5 years. During the mean follow-up of 3.13 ± 0.94 years, 3,919 persons developed diabetes. The mean eGFR was 110.44 ± 15.13 mL/min·1.73 m^2^, and the mean BMI, FPG, DBP, and SBP were 23.23 ± 3.34 kg/m^2^, 4.91 ± 0.61 mmol/L, 74.13 ± 10.78 mmHg, and 118.92 ± 16.31 mmHg, respectively. A number of participants with missing TG, TC, LDL-C, and HDL-C values were 3,119 (1.564%), 3,114 (1.561%), 83,314 (41.775%), and 84,402 (42.321%), respectively. Besides, the missing values of SBP, DBP, AST, and ALT were 19 (0.010%), 20 (0.010%), 115,231 (57.779%), and 1,110 (0.557%), respectively. In addition, the missing values of drinking and smoking status were 142,038 (71.220%) and 142,038 (71.220%). According to the inclusion and exclusion criteria, there were no missing values for other variables, such as age, BMI, and FPG.

### Baseline Characteristics of the Study Participants

Table 1 shows the baseline characteristics according to eGFR groups. We divided participants into subgroups according to eGFR levels (<90, 90–104.5, 104.5–114.5, 114.5–122.9, ≥122.9). In the highest eGFR group, the results showed that participants had lower BMI, age, blood pressure levels (including diastolic and systolic blood pressures), FPG, TG, TC, LDL-C, AST, ALT, and lower rates of current and ever drinker and smoker. Besides, in the top eGFR group, the persons had higher HDL-C levels. In addition, the group (eGFR>122.9 mL/min·1.73 m^2^) had a higher proportion of women.

**Table 1:** The baseline characteristics of participants.

**eGFR group**	**<90**	**90–104.5**	**104.5–114.5**	**114.5–22.9**	**≥122.9**	***p*** **-value**
Participants	21,250	44,544	44,538	44,507	44,596	
Age (years)	56.3 ± 14.4	49.4 ± 13.2	43.4 ± 9.3	37.2 ± 6.0	31.5 ± 4.4	<0.001
Gender						<0.001
Male	14,555 (68.5%)	28,921 (64.9%)	25,863 (58.1%)	22,840 (51.3%)	17,511 (39.3%)	
Female	6,695 (31.5%)	15,623 (35.1%)	18,675 (41.9%)	21,667 (48.7%)	27,085 (60.7%)	
BMI (kg/m^2^)	24.3 ± 3.1	23.9 ± 3.2	23.5 ± 3.2	22.9 ± 3.3	22.1 ± 3.4	<0.001
SBP (mmHg)	126.5 ± 18.8	122.7 ± 17.2	119.0 ± 15.7	115.9 ± 14.6	114.4 ± 14.0	<0.001
DBP (mmHg)	77.5 ± 11.4	76.2 ± 11.0	74.9 ± 10.9	72.8 ± 10.2	71.0 ± 9.7	<0.001
FPG (mmol/L)	5.1 ± 0.6	5.0 ± 0.6	4.9 ± 0.6	4.8 ± 0.6	4.8 ± 0.5	<0.001
eGFR (mL/min·1.73 m^2^)	81.6 ± 6.6	98.2 ± 4.1	109.8 ± 2.9	118.9 ± 2.4	128.6 ± 4.5	<0.001
TC (mmol/L)	5.0 ± 0.9	4.9 ± 0.9	4.8 ± 0.9	4.6 ± 0.8	4.4 ± 0.8	<0.001
TG (mmol/L)	1.3 (0.9, 1.9)	1.2 (0.9,1.8)	1.1 (0.8, 1.7)	1.0 (0.7,1.5)	0.9 (0.6, 1.2)	<0.001
HDL (mmol/L)	1.3 ± 0.3	1.4 ± 0.3	1.4 ± 0.3	1.4 ± 0.3	1.4 ± 0.3	<0.001
LDL (mmol/L)	2.9 ± 0.7	2.9 ± 0.7	2.8 ± 0.7	2.6 ± 0.6	2.5 ± 0.6	<0.001
ALT (U/L)	19.5 (14.5, 28.0)	19.8 (14.4, 28.7)	19.0 (13.4, 29.0)	17.4 (12.2, 28.0)	15.0 (11.0, 24.0)	<0.001
AST (U/L)	25.5 ± 10.0	25.0 ± 12.8	24.3 ± 12.4	23.5 ± 11.8	22.5 ± 12.0	<0.001
Smoking status						<0.001
Never smoker	15,470 (72.8%)	32,473 (72.9%)	33,938 (76.2%)	36,807 (82.7%)	39,289 (88.1%)	
Ever smoker	807 (3.8%)	1,782 (4.0%)	1,648 (3.7%)	1,647 (3.7%)	1,338 (3.0%)	
Current smoker	4,973 (23.4%)	10,289 (23.1%)	8,952 (20.1%)	6,053 (13.6%)	3,969 (8.9%)	
Drinking status						<0.001
Never drinker	17,510 (82.4%)	36,704 (82.4%)	37,189 (83.5%)	38,276 (86.0%)	40,092 (89.9%)	
Ever drinker	3,145 (14.8%)	6,593 (14.8%)	6,324 (14.2%)	5,608 (12.6%)	4,192 (9.4%)	
Current drinker	595 (2.8%)	1,247 (2.8%)	1,025 (2.3%)	623 (1.4%)	312 (0.7%)	
Family history of diabetes						<0.001
No	20,910 (98.4%)	43,760 (98.2%)	43,489 (97.6%)	43,380 (97.5%)	43,700 (98.0%)	
Yes	340 (1.6%)	784 (1.8%)	1,049 (2.4%)	1,127 (2.5%)	896 (2.0%)	

*Values are n (%), mean ± SD or medians (quartiles)*.

*BMI, body mass index; SBP, systolic blood pressure; DBP, diastolic blood pressure; FPG, fasting plasma glucose; TC, total cholesterol; TG, triglyceride; LDL-C, low-density lipid cholesterol; HDL-C, high-density lipoprotein cholesterol; ALT, alanine aminotransferase; AST, aspartate aminotransferase; eGFR, evaluated glomerular filtration rate; Scr, serum creatinine*.

### The Incidence Rate of Incident Diabetes

Table 2 revealed that 3,919 participants developed diabetes in total. The total incidence rate of all participants was 628.73 per 100,000 person-years. Specifically, the incidence rates of the five eGFR groups were 1225.28, 1023.29, 663.90, 340.61, and 205.60 per 100,000 person-years, respectively. Compared with the lowest eGFR group, participants with high eGFR had a lower cumulative incidence (*p* < 0.001 for trend). The cumulative incidence of total incident diabetes and each eGFR group was 1.965% (1.904–2.026%), 3.779% (3.522–4.035%), 3.183% (3.020–3.346%), 2.099% (1.966–2.233%), 1.081% (0.985–1.177%), and 0.632% (0.559–0.707%), respectively.

**Table 2:** Incidence rate of incident diabetes.

**eGFR (mL/min·1.73 m^**2**^)**	**Participants (n)**	**DM events (n)**	**Cumulative incidence (95% CI)(%)**	**Per 100,000 person-year**
Total	199,435	3,919	1.965 (1.904–2.026)	628.73
<90	21,250	803	3.779 (3.522–4.035)	1,225.28
90–104.5	44,544	1,418	3.183 (3.020–3.346)	1,023.29
104.5–114.5	44,538	935	2.099 (1.966–2.233)	663.90
114.5–122.9	44,507	481	1.081 (0.985–1.177)	340.61
≥122.9	44,596	282	0.632 (0.559–0.707)	205.60
*P* for trend			<0.001	

### Univariate Analysis

Results of the univariate analysis for the entire participants are shown in Table 3. The results showed that BMI, age, DBP, SBP, TG, LDL, TC, FPG, AST, ALT, drinking and smoking status, and family history of diabetes were positively associated with incident diabetes. In contrast, HDL-C and eGFR were negatively related to the risk of diabetes. Besides, we found that women have a lower diabetes risk than men.

**Table 3:** The results of univariate analysis.

	**Statistics**	**HR (95% CI)**	***p*** **-value**
Age (years)	42.064 ± 12.530	1.066 (1.064, 1.069)	<0.00001
**Gender**			
Male	109,690 (55.000%)	Ref.	
Female	89,745 (45.000%)	0.476 (0.444, 0.511)	<0.00001
BMI (Kg/m^2^)	23.235 ± 3.339	1.237 (1.228, 1.246)	<0.00001
SBP (mmHg)	118.923 ± 16.309	1.039 (1.037, 1.041)	<0.00001
DBP (mmHg)	74.126 ± 10.783	1.047 (1.044, 1.049)	<0.00001
eGFR (mL/min·1.73 m^2^)	110.438 ± 15.109	0.964 (0.962, 0.966)	<0.00001
FPG (mmol/L)	4.913 ± 0.612	10.494 (10.035, 10.974)	<0.00001
TC (mmol/L)	4.711 ± 0.898	1.429 (1.387, 1.472)	<0.00001
TG (mmol/L)	1.341 ± 1.032	1.264 (1.253, 1.276)	<0.00001
HDL-C (mmol/L)	1.368 ± 0.307	0.516 (0.464, 0.574)	<0.00001
LDL-C (mmol/L)	2.716 ± 0.681	1.475 (1.414, 1.539)	<0.00001
ALT (U/L)	24.011 ± 22.077	1.005 (1.004, 1.005)	<0.00001
AST (U/L)	24.000 ± 12.088	1.008 (1.007, 1.008)	<0.00001
**Smoking status**			
Never smoker	157,964 (79.206%)	Ref.	
Ever smoker	7,245 (3.633%)	1.658 (1.397, 1.968)	<0.00001
Current smoker	34,226 (17.161%)	2.059 (1.913, 2.216)	<0.00001
**Drinking status**			
Never drinker	169,771 (85.126%)	Ref.	
Ever drinker	25,829 (12.951%)	1.301 (1.180, 1.435)	<0.00001
Current drinker	3,835 (1.923%)	2.559 (1.904, 3.439)	<0.00001
**Family history of diabetes**			
No	195,239 (97.896%)	Ref.	
Yes	4,196 (2.104%)	1.695 (1.448, 1.984)	<0.00001

**Figure 1:**
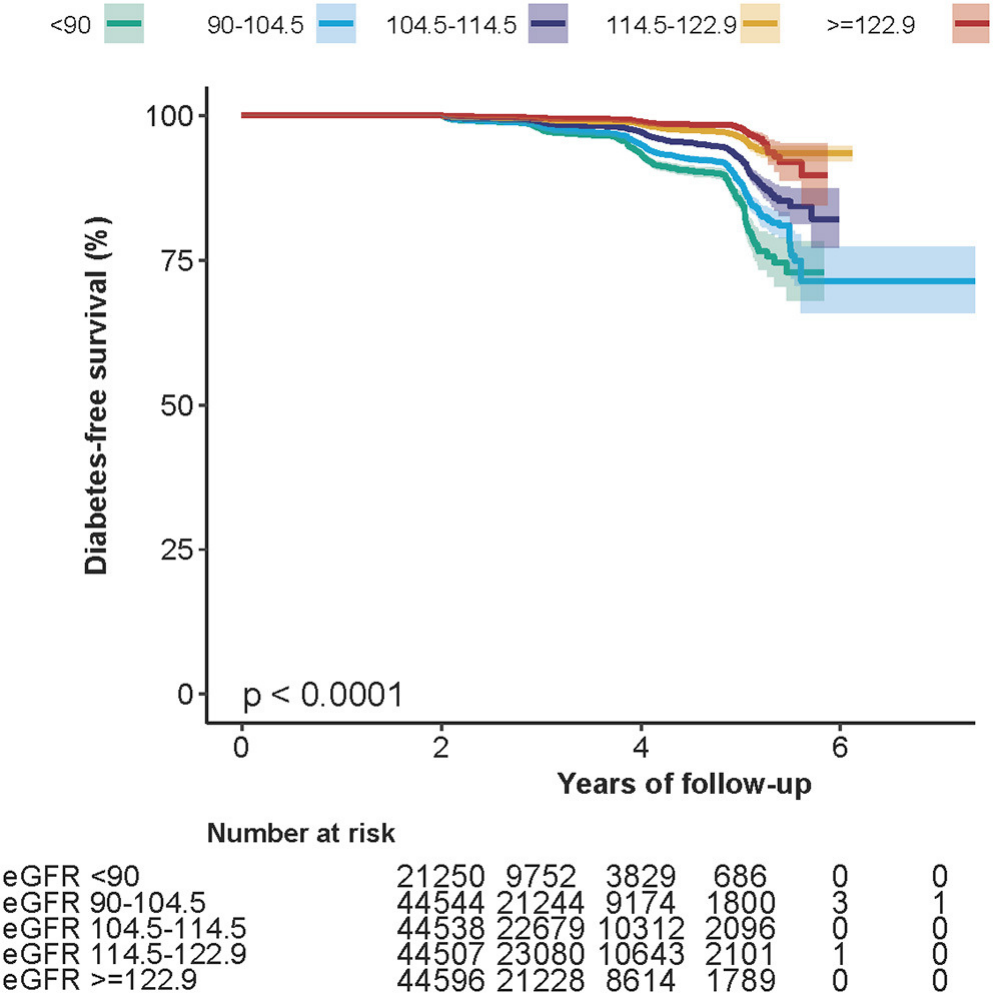
Kaplan–Meier event-free survival curve. Kaplan–Meier analysis of incident diabetes-free survival based on eGFR groups (log-rank, *p* < 0.0001).

Kaplan–Meier survival curves for the probability of diabetes-free survival stratified by eGFR groups are shown in Figure 1. The probability of diabetes-free survival between eGFR groups was significantly different (log-rank test, *p* < 0.0001). As the group of eGFR increased, the probability of diabetes-free survival gradually increased, indicating the top group with the lowest diabetes risk.

### The Results of the Relationship Between EGFR and Incident Diabetes

We used Cox proportional hazards regression model to explore the associations between eGFR and incident diabetes. Meanwhile, we showed the non-adjusted and two adjusted models in Table 4. In crude model, eGFR was negatively associated with incident diabetes [HR = 0.964, 95% confidence interval (CI):0.962–0.966, *p* < 0.00001]. In the minimally adjusted model (adjusted BMI, gender, DBP, SBP, smoking and drinking status, and family history of diabetes), the result did not change significantly (HR: 0.977, 95% CI: 0.975–0.979). After adjusting for the full model (adjusted BMI, gender, DBP, SBP, TC, LDL, TG, HDL, FPG, AST, ALT, family history of diabetes, and smoking and drinking status), we found that the relationship still exists (HR = 0.986, 95% CI: 0.984–0.988, *p* < 0.00001). The results showed that for every 1 mL/min·1.73 m^2^ increased in eGFR, the risk of diabetes decreased by 1.4%.

**Table 4:** Relationship between eGFR and the incident diabetes in different models.

**Exposure**	**Crude model (HR, 95% CI, *p*)**	**Adjust I (HR, 95% CI, *p*)**	**Adjust II (HR, 95% CI, *p*)**
eGFR	0.964 (0.962, 0.966) <0.00001	0.977 (0.975, 0.979) <0.00001	0.986 (0.984, 0.988) <0.00001
**eGFR group**			
<90	Ref.	Ref.	Ref.
90–104.5	0.779 (0.714, 0.849) <0.00001	0.908 (0.832, 0.990) 0.0294	0.986 (0.903, 1.077) 0.7595
104.5–114.5	0.480 (0.437, 0.528) <0.00001	0.663 (0.603, 0.730) <0.00001	0.770 (0.698, 0.848) <0.00001
114.5–122.9	0.244 (0.218, 0.274) <0.00001	0.398 (0.355, 0.447) <0.00001	0.590 (0.524, 0.664) <0.00001
≥122.9	0.160 (0.140, 0.183) <0.00001	0.308 (0.268, 0.353) <0.00001	0.501 (0.435 0.577) <0.00001
*P* for trend	<0.00001	<0.00001	<0.00001

*Crude model: we did not adjust other covariates*.

*Model I: we adjust gender, BMI, SBP, DBP, family history of diabetes, and smoking and drinking status*.

*Model II: we adjust gender, BMI, SBP, DBP, FPG, TC, TG, HDL-C, LDL-C, ALT, AST, family history of diabetes, and smoking and drinking status*.

*CI, confidence interval, Ref, reference*.

*eGFR: (mL/min·1.73 m^2^)*.

We also treated eGFR as a categorical variable for sensitivity analysis. Compared with the lowest group (eGFR <90 mL/min·1.73 m^2^) in the full adjusted model, the risk of diabetes in the highest group (eGFR≥122.9 mL/min·1.73 m^2^) was reduced by 49.9%, and the trend of the five groups was found to be significant (*p* < 0.00001).

In other sensitivity analyses, we excluded smoking and drinking status from the multivariate model. The results suggested that after adjusting BMI, gender, DBP, SBP, TC, LDL, TG, HDL, FPG, AST, ALT, family history of diabetes, eGFR was still negatively associated with incident diabetes (HR = 0.986, 95% CI:0.983–0.988, *p* < 0.00001) (Supplementary Table 1). We also excluded participants with fasting blood glucose >6.1 mmol/L. The results suggested that after adjusting the confounding factors, eGFR was also negatively associated with incident diabetes (HR = 0.983, 95% CI:0.980–0.986, *p* < 0.00001) (Supplementary Table 2). The sensitivity analysis results showed that the relationship between eGFR and the risk of diabetes was very robust.

In addition, we used sensitivity analysis to identify whether the created complete data had significant differences from preimputation data. We found that the relationship between eGFR and incident diabetes was consistent in the five imputed datasets and the data before imputation (Supplementary Table 3).

### The Analyses of the Non-linear Relationship

**Figure 2:**
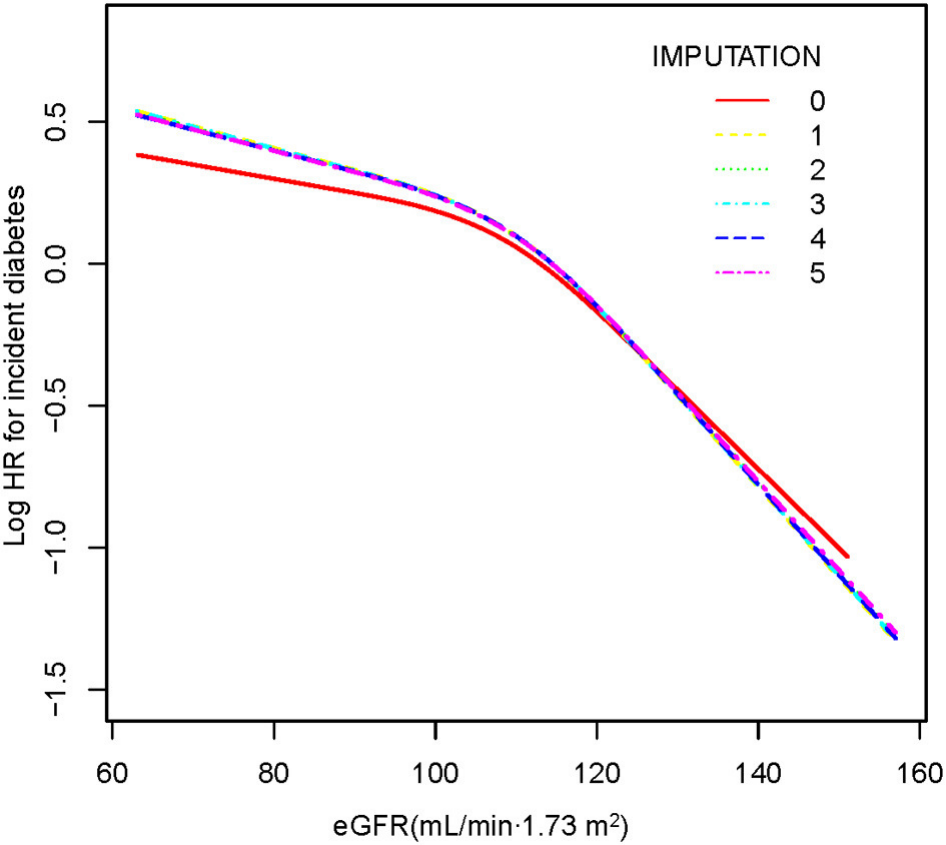
The non-linear relationship between eGFR and incident diabetes. A non-linear relationship was detected after adjusting for gender, BMI, SBP, DBP, FPG, TC, TG, HDL-C, LDL-C, ALT, AST, family history of diabetes, and smoking and drinking status. There were no significant differences between preimputation data and imputed datasets.

The Cox proportional hazards regression with cubic spline functions and smooth curve fitting (the cubic spline smoothing) were used to explore the association between eGFR and incident diabetes (Figure 2). We found that the relationship between eGFR and diabetes risk was also non-linear (adjusted BMI, gender, DBP, SBP, TC, LDL, TG, HDL, FPG, AST, ALT, family history of diabetes, and smoking and drinking status). Using a two-piecewise linear slope model, we calculated that the eGFR inflection point was 98.034 mL/min·1.73 m^2^ (log-likelihood ratio test *p* < 0.001). On the left of the inflection point, we found a tiny negative relationship between eGFR and incident diabetes (HR:0.998, 95% CI: 0.993–1.003, *p* = 0.4615). However, we observed an apparent negative relationship between eGFR and incident diabetes (HR:0.976, 95% CI: 0.972–0.980, *p* < 0.0001) on the right side of the inflection point. Among those above the inflection point, a 2.4% decrease in relative risk of diabetes was associated with a 1 mL/min·1.73 m^2^ increase in eGFR. We also found that the absolute risk of diabetes decreased by 0.07% with every 1 mL/min·1.73 m^2^ increase in eGFR (Table 5). It was apparent from the figure that the non-linear association between the eGFR and incident diabetes was stable before and after multiple imputations.

**Table 5:** The result of the two-piecewise linear regression model.

	**Incident diabetes**	**Risk**
	**(HR, 95% CI, *p*)**	**difference (%)**
Fitting model by standard linear regression	0.986 (0.984, 0.988) <0.0001	−0.06
**Fitting model by two-piecewise linear regression**		
Inflection point of eGFR	98.034	
≤ 98.034	0.998 (0.993, 1.003) 0.4615	−0.04
> 98.034	0.976 (0.972, 0.980) <0.0001	−0.07
P for log-likelihood ratio test	<0.001	

*CI, Confidence interval; RD, risk difference*.

*We adjusted gender, BMI, SBP, DBP, FPG, TC, TG, HDL-C, LDL-C, ALT, AST, family history of diabetes, and smoking and drinking status*.

### The Results of Subgroup Analyses

We used subgroup analysis to see other potential risks in the association between eGFR and diabetes events to assess factors that might affect the outcome. We treated gender, age, BMI, DBP, SBP, FPG, smoking and drinking status, and family history of diabetes as the stratification variables to detect the trend of effect sizes in these variables (Table 6). We first tested the proportion hypothesis and found that separate Cox PH models were fit within each subgroup. We noticed that many interactions were observed according to our prior norms, including FPG, gender, BMI, SBP, DBP, family history of diabetes, and smoking status (all *p*-values for interaction < 0.05). In this study, a stronger association was observed in the population with BMI <24 kg/m^2^, FPG <6.1 mmol/L, DBP <90 mmHg, SBP <140 mmHg, family history without diabetes, and never smokers. Moreover, we could also find a stronger association between eGFR and incident diabetes in women. In contrast, the weaker association was probed in men, current smokers, current and ever drinkers, and the population with BMI≥28 kg/m^2^, FPG≥6.1 mmol/L, DBP≥90 mmHg, SBP≥140 mmHg and family history with diabetes.

**Table 6:** Effect size of eGFR on incident diabetes in prespecified and exploratory subgroups.

**Characteristic**	**No of participants**	**HR (95% CI)**	***p*** **-value**	**P for interaction**
Age (years)				0.8575
20 to <30 30 to <40 40 to <50 50 to <60 60 to <70 ≥70	26,726 78,227 42,909 28,388 16,795 6,390	0.999 (0.979, 1.020) 1.010 (1.002, 1.018) 1.005 (0.999, 1.012) 1.002 (0.997, 1.008) 1.003 (0.996, 1.009) 1.009 (0.999, 1.018)	0.9392 0.0137 0.1250 0.3843 0.3952 0.0723	
Gender				<0.0001
Male	109,690	0.988 (0.986, 0.991)	<0.0001	
Female	89,745	0.981 (0.977, 0.985)	<0.0001	
BMI (Kg/m^2^)				<0.0001
<18.5	11,367	0.969 (0.946, 0.992)	0.0093	
≥18.5, <24	109,984	0.980 (0.975, 0.984)	<0.0001	
≥24, <28	61,047	0.985 (0.982, 0.989)	<0.0001	
≥28	17,037	0.997 (0.993, 1.002)	0.2412	
FPG (mmol/L)				<0.0001
<6.1	192,704	0.978 (0.975, 0.981)	<0.0001	
≥6.1	6,731	0.993 (0.990, 0.996)	<0.0001	
Smoking status				<0.0001
Never smoker	157,964	0.983 (0.981, 0.986)	<0.0001	
Ever smoker	7,245	0.986 (0.975, 0.998)	0.0185	
Current smoker	34,226	0.993 (0.988, 0.998)	0.0031	
Drinking status				<0.0001
Never drinker	169,771	0.985 (0.982, 0.987)	<0.0001	
Ever drinker	25,829	0.989 (0.982, 0.996)	0.0015	
Current drinker	3,835	1.000 (0.985, 1.016)	0.9596	
Family history of diabetes				<0.0001
No	195,239	0.985 (0.983, 0.987)	<0.000	1
Yes	4,196	0.999 (0.988, 1.011)	0.9449	
SBP				<0.0001
<140	179,683	0.984 (0.981, 0.986)	<0.0001	
≥140	19,752	0.993 (0.989, 0.997)	0.0003	
DBP				<0.0001
<90	183,590	0.984 (0.981, 0.986)	<0.0001	
≥90	15,845	0.998 (0.993, 1.003)	0.4429	

*Above model adjusted for gender, BMI, SBP, DBP, FPG, TC, TG, HDL-C, LDL-C, ALT, AST, family history of diabetes, smoking and drinking status*.

*In each case, the model is not adjusted for the stratification variable*.

*The p-value of individual PH in each subgroup was >0.05*.

## Discussion

The present retrospective cohort study showed that eGFR was negatively associated with incident diabetes after adjusting some covariates (Cox proportional hazards models). Furthermore, the trend of the effect sizes was inconsistent [left (HR:0.998, 95% CI: 0.993–1.003, *p* = 0.4615); right (HR: 0.976, 95% CI: 0.972–0.980, *p* < 0.0001)] on the left and right sides of the inflection point. The results indicated a non-linear relationship on the association between eGFR and new-onset diabetes. Subgroup analysis showed a stronger association in women, never smokers, and the population with BMI <24 kg/m^2^, FPG <6.1 mmol/L, DBP <90 mmHg, SBP <140 mmHg, and family history without diabetes. In contrast, the weaker association was probed in men, current smokers, current and ever drinkers, and the population with BMI ≥28 kg/m^2^, FPG≥6.1 mmol/L, DBP≥90 mmHg, SBP≥140 mmHg, and family history with diabetes.

Some previous studies have explored the association between eGFR and the risk of diabetes. However, most of these studies were not conducted in the Chinese population (6, 7, 23, 24). In one of such studies with 864 adults in the USA, C. Lorenzo et al. (7) found that the association between glomerular filtration rate and incident diabetes was not linear, which indicated that individuals with the upper and lower limits of GFR had an increased risk of diabetes in the future. GFR and type 2 diabetes might have a common pathogenic mechanism. Some other studies have explored the association between CKD and incident diabetes. A prospective cohort study focused on 1,713 American participants with reduced GFR and no diabetes at baseline found that the incidence of T2DM in individuals with CKD was significantly higher than that in the general population (25). Another population-based cohort study in Taiwan found that CKD was an essential and independent predictor of diabetes (adjusted HR 1.204; 95% CI 1.11, 1.31) (26). We obtained the same results through the Cox proportional hazards regression model, showing that eGFR was negatively associated with the incidence of diabetes. In addition, our research had a larger sample size (199,435) from 32 locations and 11 cities in China and was more representative of the Chinese population.

In contrast to the results of these studies, eGFR could not predict diabetes risk in one study with 1,337,452 veterans conducted in the USA (23). Researchers found that every 10 ml/min/1.73 m^2^ reduction in eGFR had nothing to do with the risk of developing diabetes (1.00; 1.00–1.01). A similar study in a lean, normoglycemic healthy women population in Israel showed that in a logistic regression model adjusted for BMI, age, smoking, socioeconomic status, serum uric acid, and baseline glucose, eGFR was associated with an increased risk of developing diabetes (1.02; 1.01–1.03) (24). We compared these studies mentioned above, and the inconsistent results might come from the following: (1) the study population was different. These studies that were inconsistent with our results mainly focused on Israel and the USA. (2) Many studies with those different conclusions did not clearly clarify the non-linear relationship and used different regression models. (3) Compared with our research, those studies did not consider the effect of DBP, SBP, TG, TC, LDL, HDL, AST, ALT, family history of diabetes, and drinking status on the association between eGFR and incident diabetes when adjusting covariates. However, previous studies considered these variables as the factors related to eGFR or diabetes risk. (4) This might be related to different kidney functions. Some studies have shown that eGFR and insulin resistance or incident diabetes association differ between different CKD stages (6, 27, 28).

This study found that using Cox proportional hazards regression with cubic spline functions and smooth curve fitting (the cubic spline smoothing) to show a non-linear relationship was different from that obtained by Lorenzo et al. (7). Their study used subgroup analysis stratified by GFR categories to assess a U-shaped relation between eGFR and risk of T2DM. They found that individuals within the upper and lower ranges of the GFR have an increased risk of diabetes in the future. In contrast, in this study, we found that the association between eGFR and incident diabetes was not obvious when eGFR was <98.034 mL/min·1.73 m^2^. However, we observed an apparent negative relationship between eGFR and incident diabetes on the right side of the inflection point. Differences might be caused by race, levels of renal function, and different methods of evaluating GFR. The GFR was estimated using the Modification of Diet in Renal Disease (MDRD) study equation based on six variables in their study (29, 30). Since the participants in our study were all with eGFR above 60 ml/min/1.73m^2^, we used the CKD-EPI equation (9) to estimate GFR, leading to a more precise GFR estimation for Chinese patients with CKD in general practice, especially in the patients with higher GFR. The eGFR levels of the participants in their study were in the range of 39.9–239.1. Additionally, they found that the risk of T2DM increased in participants with eGFR below 65 and above 100 ml min^−1^ 1.73 m^−2^. The range of eGFR in this study was 63.0–157.0 ml min^−1^ 1.73 m^−2^. In addition, we found a negative association between eGFR and risk of diabetes in participants with eGFR above 98.034 ml min^−1^ 1.73 m^−2^. A total of 283 (32.8%) participants had IGT in their study, whereas in this study, only 5,181 (2.6%) persons with FPG had above 6.1 mmol/L. Therefore, in their research, the results indicated that the highest eGFR values were also associated with an increased risk of diabetes. This might be related to ultrafiltration.

According to the study by Lorenzo et al. (7), when eGFR was lower than 80 ml/min, the association between eGFR and diabetes risk was also non-linear. The study showed that with the increase of eGFR, the cumulative probability of diabetes first increases and then decreases. Some other studies suggested that the reduced eGFR was associated with an increased cumulative probability of diabetes when eGFR(baseline or time-updated) was <60 ml/min (23, 25). Another study found that, compared with eGFR>90 ml/min, each 10 ml/min per 1.73 m^2^ lower eGFR was associated with a 2.2% higher fasting insulin concentration (95% CI, 1.4%, 2.9%; *p* < 0.001) and a 1.1% lower insulin sensitivity index (95% CI, 0.03%, 2.2%; *p* = 0.04). Surprisingly, reduced eGFR was associated with an augmented B cell function index (*p* < 0.001), lower 2-h glucose concentration (*p* = 0.002), and decreased risk of glucose intolerance (*p* = 0.006) (6). In this study, we found that there was no relationship between eGFR and incident diabetes when eGFR was in the range of 60–98.034 ml/min per 1.73 m^2^.

Based on the above-related literature reports, we considered that this phenomenon might be related to the decrease in eGFR, leading to an increased risk of diabetes and a decreased insulin sensitivity. Still, it was also associated with the reduction in eGFR leading to increased insulin levels and augmented B cell function in the body. As the mechanisms between eGFR and glucose sensitivity are complicated, those with truly impaired eGFR have other underlying pathologies and are inherently different than those with normal kidney function. So, further research is still needed. The results would be helpful for future research on establishing diagnostic or predictive models of the risk of diabetes.

In recent years, researchers have clarified the relationship between GFR and insulin resistance. In a community-based cohort study, the results suggested that insulin sensitivity measured with euglycemic clamp was independently related to eGFR, and impaired insulin sensitivity might be associated with the development of early renal dysfunction before the onset of diabetes (28). In another community-based cohort study in US older adults, researchers found that lower eGFR was related to insulin resistance. However, as eGFR decreased, impaired glucose tolerance and the risk of diabetes did not increase (6). Disturbances in insulin homeostasis and glucose in patients with CKD are complex and represent two opposite effects. On the one hand, CKD reduces insulin sensitivity (and increases insulin resistance) and leads to β-cell dysfunction and insulin secretion defects in the late stage (31). On the other hand, CKD leads to a decrease in insulin clearance, thereby prolonging its half-life (32, 33). The balance of these two opposing forces determines glucose metabolism and ultimately determines the risk of any individual suffering from diabetes.

Our study has some strengths. (1) Compared to other similar studies, the present sample size was relatively large; as it exceeded most sample sizes of similar studies; (2), we expounded the non-linear relationship and found the inflection point; (3) this study was observational, so it is likely to cause potential confusion. The strict statistical adjustment was used to minimize residual confounders; (4) a series of sensitivity analysis and subgroup analysis were performed to assess the reliability of our results; (5) to control bias, we did not exclude missing values for covariates, and we used multiple multivariate imputations to handle the missing data of covariants and included them in the Cox proportional hazards regression models.

There are still some potential limitations. First, the raw data were from the Chinese population, which limited the generalizability of our findings. In addition, since this was a secondary analysis study, the information did not contain other relevant factors, such as medication history, socioeconomic factors, etc. We could not adjust those variables. Similarly, we could not distinguish among type 1, type 2, and other types of diabetes. Second, they did not perform a 2-h oral glucose tolerance test or a glycosylated hemoglobin determination. According to 1999 WHO recommendations for the diagnosis of diabetes, the definition of diabetes in our study might lead to missing some diabetic patients (34). However, oral glucose tolerance tests and glycosylated hemoglobin determination were not feasible in such a large cohort. Third, we only measured eGFR and other parameters at baseline did not consider changes of eGFR over time. In the future, we can consider designing our studies or collaborating with other researchers to collect as many variables as possible, including information on the evolution of renal function during patients follow-up. We would also perform an oral glucose tolerance test and determine glycosylated hemoglobin to diagnose diabetes more accurately. Third, the information from the original study did not include albuminuria, censoring by death during follow-up, and observed cases of diabetes during the first 2 years of follow-up. For such a large sample of participants, possible deaths during follow-up and diabetes observed in the first 2 years were inevitable. In the future, we can consider designing our studies and collecting albuminuria, the potential censoring by death, and the observed cases of diabetes throughout the follow-up period. Therefore, we could analyze the relationship between eGFR and diabetes through a competitive risk model in patients with unnecessary degrees of kidney damage. Finally, although we have adjusted several confounding factors to the possible influences, residual confounding may exist and further investigations are needed.

## Conclusion

Evaluated glomerular filtration rate is independently associated with incident diabetes. The relationship between eGFR and incident diabetes is also non-linear. eGFR is obvious negatively related to incident diabetes when eGFR is above 98.034 mL/min·1.73 m^2^. In addition, a stronger association of eGFR and incident diabetes was detected in women, never smokers, and the population with BMI <24 kg/m^2^, FPG <6.1 mmol/L, DBP <90 mmHg, SBP <140 mmHg, and family history without diabetes. This study provides a further reference for the prevention of diabetes in patients with different renal function states.

## Data Availability Statement

The original contributions presented in the study are included in the article/Supplementary Material, further inquiries can be directed to the corresponding author/s.

## Ethics Statement

The studies involving human participants were reviewed and approved by the Rich Healthcare Group Review Board. The patients/participants provided their written informed consent to participate in this study.

## Author Contributions

ZM and HH contributed to the study concept and design, researched and interpreted the data, and drafted the manuscript. XD, QH, and PC oversaw the project's progress, contributed to the discussion, and reviewed the manuscript. LL and ZY revised the manuscript. HH and ZY are the guarantors of this work and as such have full access to all the data in the study and take responsibility for the integrity of the data and the accuracy of the data analysis. All authors read and approved the final manuscript.

## Funding

This study was supported by the Discipline Construction Ability Enhancement Project of Shenzhen Health Commission (SZXJ2017031).

## Conflict of Interest

The authors declare that the research was conducted in the absence of any commercial or financial relationships that could be construed as a potential conflict of interest.

## Publisher's Note

All claims expressed in this article are solely those of the authors and do not necessarily represent those of their affiliated organizations, or those of the publisher, the editors and the reviewers. Any product that may be evaluated in this article, or claim that may be made by its manufacturer, is not guaranteed or endorsed by the publisher.

## Abbreviations

DBP: Diastolic blood pressure
SBP: systolic blood pressure
BMI: body mass index
Scr: serum creatinine
eGFR: evaluated glomerular filtration rate
FPG: fasting plasma glucose
TG: triglyceride
TC: total cholesterol
LDL-C: low-density lipid cholesterol
HDL-C: high-density lipoprotein cholesterol
AST: aspartate aminotransferase
ALT: alanine aminotransferase
GAM: generalized additive models
T2DM: type 2 diabetes mellitus
CKD: chronic kidney disease
HR: hazard ratios
CI: confidence intervals
RD: risk difference.
