# Supplementary material for: Association of Evaluated Glomerular Filtration Rate and Incident Diabetes Mellitus: A Secondary Retrospective Analysis Based on a Chinese Cohort Study
Source: Front Med (Lausanne). 2022 Jan 31;8:724582. doi: 10.3389/fmed.2021.724582 (PMC8841619; doi:10.3389/fmed.2021.724582)
Supplement: Supplementary file 2 [file Table_2.DOCX]

**Association of evaluated glomerular filtration rate and incident diabetes mellitus: a secondary retrospective analysis based on a Chinese cohort study**

**Running title: eGFR and incident diabetes**

**Zihe Mo^1#^, Haofei Hu^2,3#^, Xiaoqing Du^1^,Qingli Huang^1^, Ping Chen^1^, Linjing Lai^1^, Zhiqun Yu^1*^**

^1^Department of Physical Examination, DongGuan Tungwah Hospital, Dongguan 523000, Guangdong Province, China

^2^Department of Nephrology,The First Affiliated Hospital of Shenzhen University, Shenzhen 518035, Guangdong Province, China

^3^Department of Nephrology, Shenzhen Second People’s Hospital, Shenzhen 518035, Guangdong Province, China

Zihe Mo and Haofei Hu have contributed equally to this work.

*Corresponding author

Zhiqun Yu

Department of Physical Examination, DongGuan Tungwah Hospital,

No. 1 Dongcheng Road, Dongcheng Street,

Dongguan 523000,

Guangdong Province,

China

E-mail: [yuzhiqun12345@126.com](mailto:yuzhiqun12345@126.com)

**TableS1** **Relationship between eGFR and the incident diabetes in different models without adjusting smoking and drinking status**

| Exposure | Crude model (HR,95%CI,P) | Adjust I(HR,95%CI,P) | Adjust II(HR,95%CI,P) |
| --- | --- | --- | --- |
| eGFR | 0.964 (0.962, 0.966) <0.00001 | 0.976 (0.974, 0.978) <0.00001 | 0.986 (0.983, 0.988) <0.00001 |
|  |  |  |  |
| eGFR group |  |  |  |
| <90 | Ref. | Ref. | Ref. |
| 90-104.5 | 0.779 (0.714, 0.849) <0.00001 | 0.913 (0.837, 0.996) 0.04050 | 0.990 (0.907, 1.080) 0.81490 |
| 104.5-114.5 | 0.480 (0.437, 0.528) <0.00001 | 0.665 (0.604, 0.732) <0.00001 | 0.771 (0.700, 0.849) <0.00001 |
| 114.5-122.9 | 0.244 (0.218, 0.274) <0.00001 | 0.391 (0.348, 0.439) <0.00001 | 0.584 (0.519, 0.657) <0.00001 |
| ≥122.9 | 0.160 (0.140, 0.183) <0.00001 | 0.300 (0.261, 0.344) <0.00001 | 0.495 (0.429, 0.570) <0.00001 |
| P for trend | <0.00001 | <0.00001 | <0.00001 |

Crude model: we did not adjust other covariates.

Model I: we adjust gender, BMI, SBP, DBP, family history of diabetes.

Model II: we adjust gender, BMI, SBP, DBP, FPG, TC, TG, HDL-C, LDL-C, ALT, AST, family history of diabetes

CI: confidence interval, Ref: reference.

**TableS2 Relationship between eGFR and the incident diabetes in different models after excluding participants with FPG>6.1mmol/L**

| Exposure | Crude model (HR,95%CI,P) | Adjust I(HR,95%CI,P) | Adjust II(HR,95%CI,P) |
| --- | --- | --- | --- |
| eGFR | 0.966 (0.964, 0.969) <0.00001 | 0.979 (0.976, 0.981) <0.00001 | 0.983 (0.980, 0.986) <0.00001 |
|  |  |  |  |
| eGFR group |  |  |  |
| <90 | Ref. | Ref. | Ref. |
| 90-104.5 | 0.786 (0.697, 0.886) 0.00009 | 0.904 (0.801, 1.020) 0.10159 | 0.902 (0.799, 1.019) 0.09734 |
| 104.5-114.5 | 0.509 (0.447, 0.579) <0.00001 | 0.693 (0.608, 0.790) <0.00001 | 0.719 (0.630, 0.822) <0.00001 |
| 114.5-122.9 | 0.276 (0.238, 0.320) <0.00001 | 0.440 (0.378, 0.512) <0.00001 | 0.519 (0.445, 0.605) <0.00001 |
| ≥122.9 | 0.193 (0.163, 0.230) <0.00001 | 0.364 (0.305, 0.434) <0.00001 | 0.463 (0.387, 0.555) <0.00001 |
| P for trend | <0.00001 | <0.00001 | <0.00001 |

Crude model: we did not adjust other covariates.

Model I: we adjust gender, BMI, SBP, DBP, family history of diabetes, smoking and drinking status.

Model II: we adjust gender, BMI, SBP, DBP, FPG, TC, TG, HDL-C, LDL-C, ALT, AST, family history of diabetes, smoking and drinking status.

CI: confidence interval, Ref: reference.

**TableS3 Relationship between eGFR and the incident diabetes in pre-imputation data and imputed datasets**

| Exposure | pre-imputation | imputation 1 | imputation 2 | imputation 3 | imputation 4 | imputation 5 |
| --- | --- | --- | --- | --- | --- | --- |
| Non-adjusted |  |  |  |  |  |  |
| eGFR | 0.964 (0.962, 0.966) <0.00001 | 0.964 (0.962, 0.966) <0.00001 | 0.964 (0.962, 0.966) <0.00001 | 0.964 (0.962, 0.966) <0.00001 | 0.964 (0.962, 0.966) <0.00001 | 0.964 (0.962, 0.966) <0.00001 |
| eGFR group |  |  |  |  |  |  |
| <90 | Ref. | Ref. | Ref. | Ref. | Ref. | Ref. |
| 90-104.5 | 0.779 (0.714, 0.849) <0.00001 | 0.779 (0.714, 0.849) <0.00001 | 0.779 (0.714, 0.849) <0.00001 | 0.779 (0.714, 0.849) <0.00001 | 0.779 (0.714, 0.849) <0.00001 | 0.779 (0.714, 0.849) <0.00001 |
| 104.5-114.5 | 0.480 (0.437, 0.528) <0.00001 | 0.480 (0.437, 0.528) <0.00001 | 0.480 (0.437, 0.528) <0.00001 | 0.480 (0.437, 0.528) <0.00001 | 0.480 (0.437, 0.528) <0.00001 | 0.480 (0.437, 0.528) <0.00001 |
| 114.5-122.9 | 0.244 (0.218, 0.274) <0.00001 | 0.244 (0.218, 0.274) <0.00001 | 0.244 (0.218, 0.274) <0.00001 | 0.244 (0.218, 0.274) <0.00001 | 0.244 (0.218, 0.274) <0.00001 | 0.244 (0.218, 0.274) <0.00001 |
| ≥122.9 | 0.160 (0.140, 0.183) <0.00001 | 0.160 (0.140, 0.183) <0.00001 | 0.160 (0.140, 0.183) <0.00001 | 0.160 (0.140, 0.183) <0.00001 | 0.160 (0.140, 0.183) <0.00001 | 0.160 (0.140, 0.183) <0.00001 |
| P for trend | <0.00001 | <0.00001 | <0.00001 | <0.00001 | <0.00001 | <0.00001 |
| Adjust I |  |  |  |  |  |  |
| eGFR | 0.980 (0.976, 0.984) <0.00001 | 0.977 (0.975, 0.979) <0.00001 | 0.977 (0.975, 0.979) <0.00001 | 0.977 (0.975, 0.979) <0.00001 | 0.977 (0.975, 0.979) <0.00001 | 0.977 (0.975, 0.979) <0.00001 |
| eGFR group |  |  |  |  |  |  |
| <90 | Ref. | Ref. | Ref. | Ref. | Ref. | Ref. |
| 90-104.5 | 1.156 (0.975, 1.371) 0.09428 | 0.906 (0.831, 0.989) 0.02673 | 0.906 (0.831, 0.989) 0.02666 | 0.906 (0.831, 0.988) 0.02638 | 0.909 (0.834, 0.992) 0.03264 | 0.912 (0.836, 0.995) 0.03760 |
| 104.5-114.5 | 0.838 (0.694, 1.012) 0.06658 | 0.660 (0.600, 0.727) <0.00001 | 0.663 (0.603, 0.730) <0.00001 | 0.661 (0.601, 0.728) <0.00001 | 0.667 (0.606, 0.733) <0.00001 | 0.665 (0.605, 0.732) <0.00001 |
| 114.5-122.9 | 0.524 (0.422, 0.651) <0.00001 | 0.399 (0.355, 0.447) <0.00001 | 0.398 (0.354, 0.446) <0.00001 | 0.397 (0.354, 0.445) <0.00001 | 0.399 (0.356, 0.448) <0.00001 | 0.398 (0.355, 0.447) <0.00001 |
| ≥122.9 | 0.324 (0.245, 0.428) <0.00001 | 0.308 (0.269, 0.354) <0.00001 | 0.308 (0.268, 0.353) <0.00001 | 0.306 (0.267, 0.352) <0.00001 | 0.309 (0.269, 0.355) <0.00001 | 0.307 (0.268, 0.353) <0.00001 |
| P for trend | <0.00001 | <0.00001 | <0.00001 | <0.00001 | <0.00001 | <0.00001 |
| Adjust II |  |  |  |  |  |  |
| eGFR | 0.988 (0.979, 0.997) 0.01087 | 0.986 (0.984, 0.988) <0.00001 | 0.986 (0.984, 0.988) <0.00001 | 0.986 (0.984, 0.988) <0.00001 | 0.986 (0.984, 0.988) <0.00001 | 0.986 (0.984, 0.988) <0.00001 |
| eGFR group |  |  |  |  |  |  |
| <90 | Ref. | Ref. | Ref. | Ref. | Ref. | Ref. |
| 90-104.5 | 1.166 (0.807, 1.684) 0.41301 | 0.982 (0.900, 1.072) 0.68399 | 0.989 (0.906, 1.079) 0.80323 | 0.983 (0.901, 1.072) 0.69467 | 0.984 (0.902, 1.074) 0.72260 | 0.994 (0.911, 1.084) 0.88952 |
| 104.5-114.5 | 0.752 (0.501, 1.128) 0.16788 | 0.767 (0.696, 0.845) <0.00001 | 0.770 (0.699, 0.848) <0.00001 | 0.767 (0.696, 0.845) <0.00001 | 0.774 (0.703, 0.853) <0.00001 | 0.770 (0.699, 0.848) <0.00001 |
| 114.5-122.9 | 0.667 (0.414, 1.073) 0.09519 | 0.585 (0.520, 0.657) <0.00001 | 0.589 (0.524, 0.663) <0.00001 | 0.587 (0.522, 0.659) <0.00001 | 0.589 (0.524, 0.661) <0.00001 | 0.600 (0.534, 0.675) <0.00001 |
| ≥122.9 | 0.640 (0.352, 1.164) 0.14346 | 0.496 (0.431, 0.571) <0.00001 | 0.505 (0.439, 0.581) <0.00001 | 0.496 (0.431, 0.571) <0.00001 | 0.502 (0.436, 0.578) <0.00001 | 0.505 (0.438, 0.581) <0.00001 |
| P for trend | 0.00506 | <0.00001 | <0.00001 | <0.00001 | <0.00001 | <0.00001 |

HR (95% CI) Pvalue

Crude model: we did not adjust other covariates.

Model I: we adjust gender, BMI, SBP, DBP, family history of diabetes, smoking and drinking status.

Model II: we adjust gender, BMI, SBP, DBP, FPG, TC, TG, HDL-C, LDL-C, ALT, AST, family history of diabetes, smoking and drinking status.

CI: confidence interval, Ref: reference.

eGFR: **(mL/min·1.73 m^2^)**
